# Multiple brain abscesses in an extremely preterm infant and a 12-year follow up: a case report

**DOI:** 10.1186/s13052-022-01294-7

**Published:** 2022-06-16

**Authors:** Shulin Pan, Su Lin, Jing Lin, Shangqin Chen, Zhenlang Lin

**Affiliations:** 1 Department of Neonatology, The Second Affiliated Hospital & Yuying Children’s Hospital, Wenzhou Medical University, Wenzhou, 325000 People’s Republic of China; 2 Department of Pediatrics, Icahn School of Medicine at Mount Sinai, NY New York, USA

**Keywords:** Multiple brain abscesses, Extremely preterm infant, Preterm, Aspiration, Prognosis

## Abstract

**Background:**

Brain abscesses are uncommon but life-threatening in extremely preterm (EP, Gestational Age < 28 weeks) infants. The information of long-time follow-up is rare, but very few cases presented almost intact neural function after injury.

**Case presentation:**

We report the clinical course and the outcome of a 27-week preterm infant with multiple brain abscesses. The brain abscesses were detected by cranial magnetic resonance imaging (MRI) and were treated with surgical aspiration twice and a 7-week course of intravenous antibiotics. The patient had two episodes of seizure like activities at 8 and 11 years old respectively, whereas she had normal results of electroencephalogram (EEG). MRI showed encephalomalacia and periventricular leukomalacia. Otherwise, she had no obvious neurological deficits based on multiple physical examination and her intellectual quotient (IQ) was in normal range in the long-time follow-up.

**Conclusions:**

Early diagnosis of brain abscesses and appropriate therapy can improve the prognosis. Furthermore, this case report provides an example of the possible neuroplasticity of brain in EP infants.

## Background

Brain abscesses are rare in extremely preterm (EP, Gestational Age < 28 weeks) infants. Previous studies have shown that brain abscesses were usually associated with severe neurological sequelae which could be devastating [1–3]. The non-specific and subtle manifestation, difficulties in diagnosis and treatment contribute to the poor outcomes. To our knowledge, there are only a few reports about brain abscesses in EP infants and even fewer follow-up data reported [4–8].

Here we report about an EP infant with multiple brain abscesses who was treated with surgical aspiration and intravenous antibiotics and for whom a 12-year follow up was available. We believe this case could provide useful information about early diagnosis and proper treatment of brain abscesses. Furthermore, it witnesses the possible neuroplasticity of brain in EP infants.

## Case presentation

Patient was a 27-week premature female infant, a product of a 36-year-old mother via an in vitro fertilization. Patient was delivered via cesarean section for preterm premature rupture of membrane lasted for 50 h. The mother received antibiotics during her antenatal period and during cesarean section. The birth weight was 1100 g. The Apgar scores at one and five minutes were 8 and 9 respectively. The mother’s prenatal labs were normal, and mother denied any infections, radiation exposure or drug uses during the pregnancy.

**Fig. 1:**
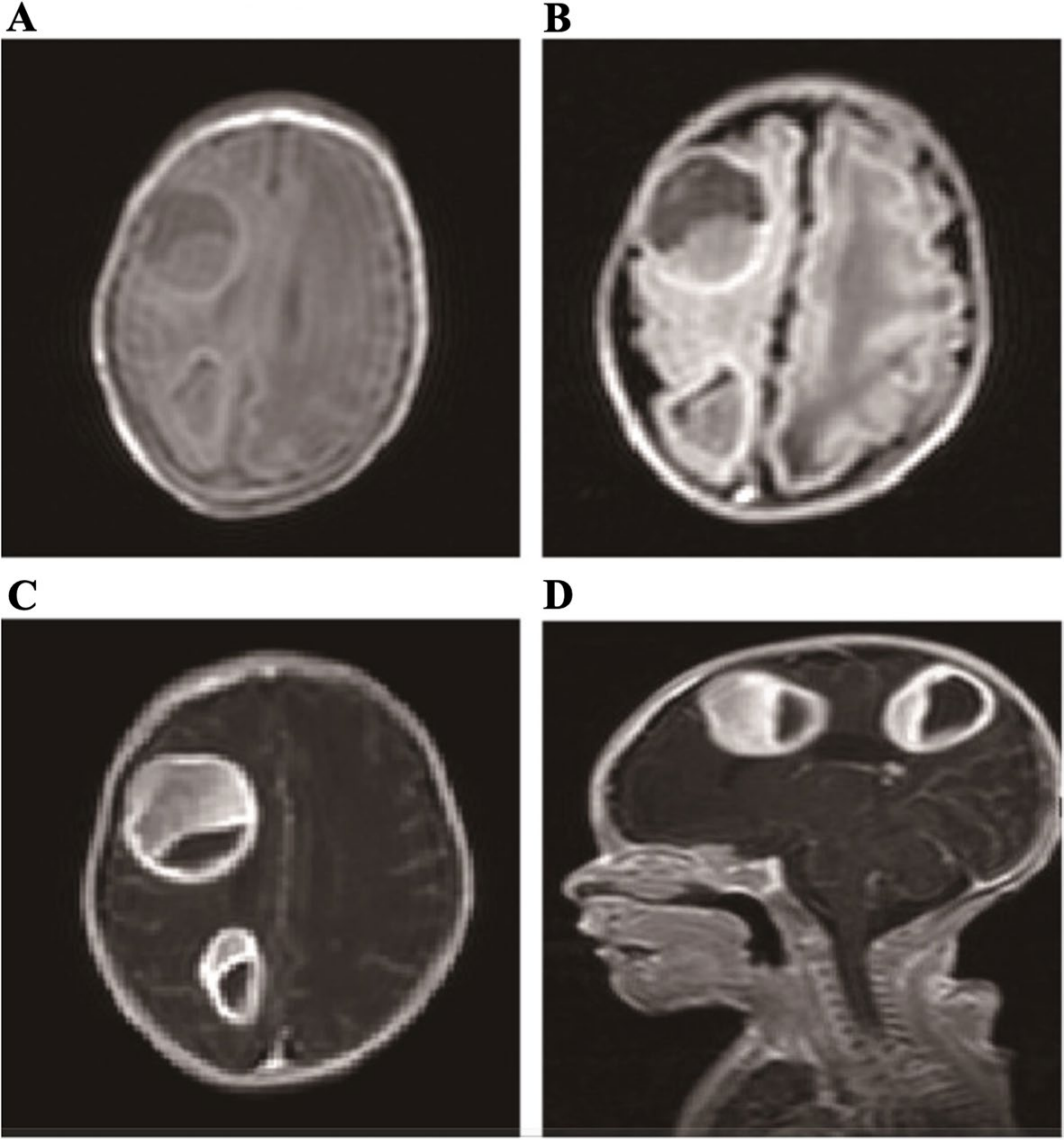
The brain revealed multiple space-occupying masses in the frontal and parietal lobes in the right brain. **A** and **B** showed the T1-weighted MRI image and T2-weighted MRI image respectively. **C** and **D** showed the T1-weighted contrast-enhanced MRI scan of the brain obtained after administration of an intravenous contrast agent

**Fig. 2:**
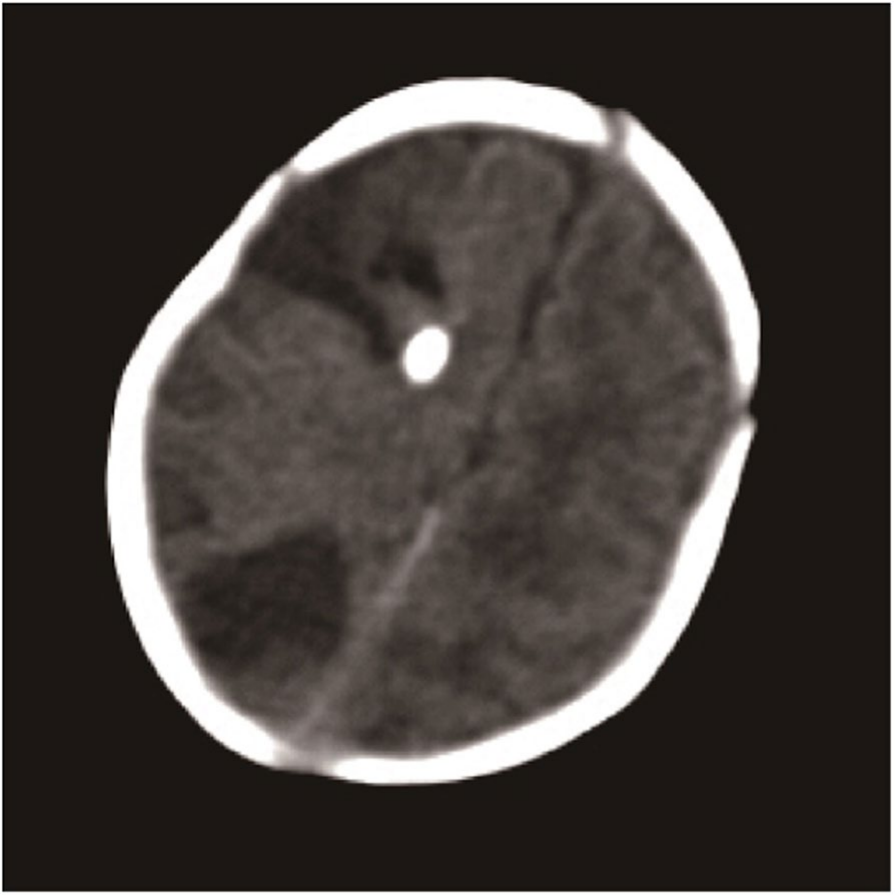
CT showed a decreased size of abscess in the frontal lobe and the hyperintensity area showed as an external ventricular drain. In the parietal area, there was an abscess with decreased attenuation. (Clarity of the figure was limited by the digital technology in our hospital in 2001)

**Fig. 3:**
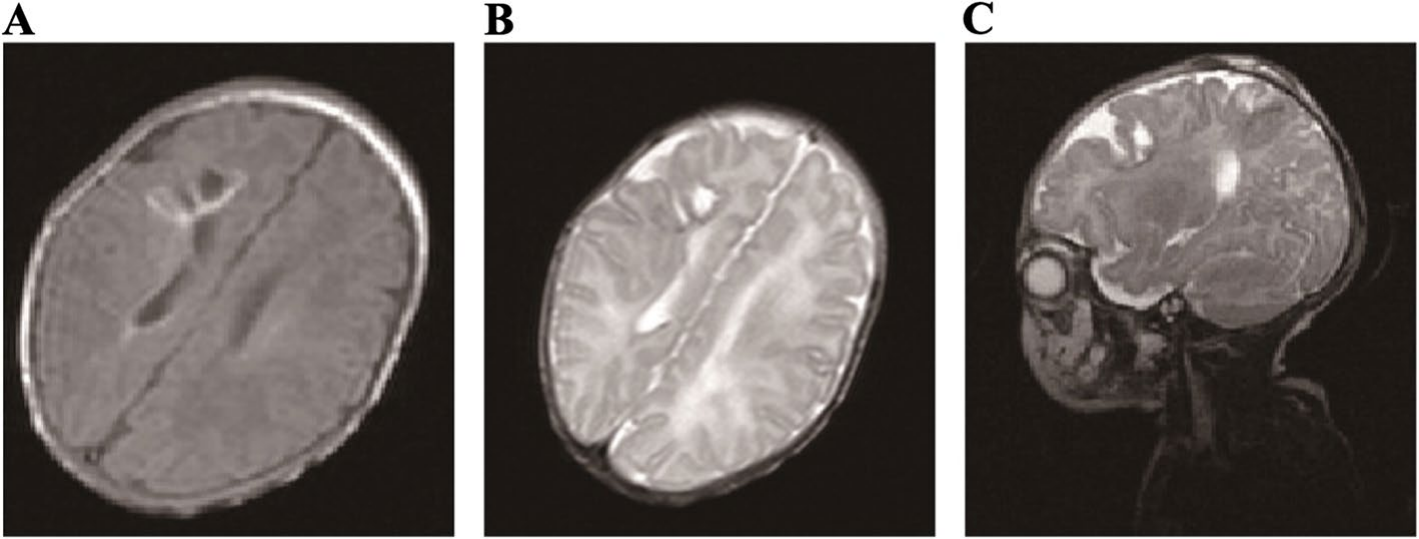
Nearly complete resolution of the abscesses with little hemorrhage. **A**, **B**, and **C** showed the T1-weighted MRI image and T2-weighted MRI image respectively

The patient was intubated on admission into neonatal intensive care unit (NICU) and received one dose of surfactant for respiratory distress syndrome. She received intravenous ampicillin and cefotaxime for seven days for suspected clinical sepsis with negative blood culture. She was successfully extubated to nasal continuous positive airway pressure (N-CPAP) on day of life 1. A peripherally inserted central catheter (PICC) was placed on day of life 1 and removed on day of life 42. On day of life 30, she was noted to have repeated apnea that required intubation and mechanical ventilation. She had a sepsis work up and was treated with a 10-day course of intravenous imipenem (10 mg/kg/dose, every 12 h) for a multi-drug resistant Klebsiella Pneumoniae growing from a culture of trachea secretion. Complete blood count showed a leukocyte count of 19.3 × 10^3^/ μ L with 68% neutrophils, 32% lymphocytes, 1.3% monocytes and 0.7% eosinophils, hemoglobin of 10.5 g / dl, and platelet count of 716 × 10^3^/ μ L. C-reactive protein increased to 15 mg/ L and decreased to normal level after administration of antibiotics for one day. The blood culture obtained before the administration of antibiotic was negative. Patient recovered and was weaned to N-CPAP again on day of life 37, and further weaned to nasal cannula oxygen therapy on day of life 41 for chronic lung disease. On day of life 55, MRI was conducted to evaluate the central nervous system due to high risk for developmental delay. MRI revealed two brain abscesses measuring 3.3 cm × 3.1 cm × 3.1 cm in diameter in the right frontal region, and another 2.5 cm × 2.1 cm × 2.6 cm in diameter in the right parietal region (Fig. 1). The brain abscesses were not communicating with the ventricles. Neurological examination revealed normal occipito-frontal circumference, reflexes, wakefulness and physiological hypotonia. Examination of other systems was normal. Congenital heart disease was ruled out by echocardiography and the arterial duct was closed. Results of complete blood count and C-reactive protein were normal. Patient was started with intravenous meropenem (40 mg/kg/dose, every eight hours) and underwent surgical aspirations with antibiotic irrigation of the abscesses twice by pediatric neurosurgeon on day of life 64 and life 83 in operating room. A total of 20 ml yellowish purulent fluids were obtained from the abscesses. The bacterial culture of the cerebral-spinal fluids and the purulent essudate were both negative for any bacterial pathogens. Purulent fluids from the right frontal region were drained for 6 days and the purulent essudate from the right parietal region was drained for 8 days after aspirations until no fluids flowing out of the drainage tubes. The follow-up computed tomography (CT) scan 5 days after the first surgical aspiration showed a decreased size of abscess in the frontal lobe (Fig. 2). Patient was treated with meropenem for a total of 7 weeks. A follow-up brain MRI scan (Fig. 3) obtained prior to discharge showed near complete resolution of the abscesses. The follow up cerebrospinal fluid examination results were normal. Patient was discharged home on day of life 106.

**Fig. 4:**
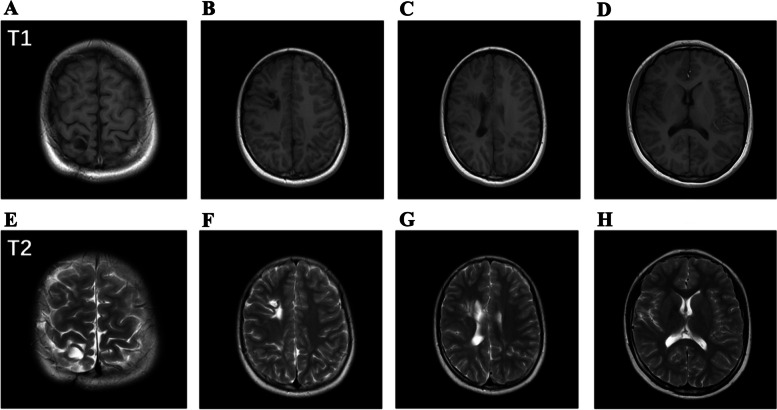
**A** and **E** showed encephalomalacia of the parietal lobe. **B** and **F** showed encephalomalacia of the frontal lobe. **C** and **G** showed that encephalomalacia in the frontal lobe of the right hemisphere was communicating with the ipsilateral lateral ventricle which dilated. **C**, **D**, **G**, and H showed the periventricular leukomalacia

At 10-month (7 months corrected age) follow-up, her weight was 8 kg. She had normal neurological examination along with normal ophthalmology and hearing evaluations. At 2-year-old, the patient was 92 cm in height (95th percentile) and her weight was 13 kg (50th percentile). The child was in good health and her parents reported no abnormal physical or behavior activities. At the age of 8 and 11, the patient had convulsions in nocturnal sleeping and the convulsions lasted for approximately three minutes with spontaneous resolution. She was admitted to the hospital twice, clinical symptoms, physical examination, laboratory screening and electroencephalography did not reveal any abnormalities. At the age of 11, MRI (Fig. 4) was conducted and the imaging finding mostly manifested as encephalomalacia in the frontal and parietal lobes of the right hemisphere. In addition, bilateral periventricular leukomalacia was observed. At the age of 12, her weight and height were in the 50th percentile and she had the test of Wechsler Intelligence Scale for Children, 4th edition (Table 1). The Full-scale IQ was 89 (23th percentile). The girl was subjected to neurocognitive evaluation with the following results: working memory (score = 100, 50th percentile), processing speed (score = 92, 30th percentile), verbal comprehension (score = 89, 23th percentile), and perceptual reasoning (score = 92, 30th percentile) were within normal range. The girl earned good grades in primary school and was particularly gifted for drawing. She did not receive rehabilitation therapy in the long-time follow-up because of normal result in every developmental milestone and she learned to swim, dance, paint, and play guitar at her early age.

**Table 1:** The result of Wechsler Intelligence Scale for Children, 4th edition at 12 years old

Cognitive test	Composite Score	Percentile Rank	95% Confidence Interval
Working Memory	100	50	93–100
Processing Speed	92	30	84–102
Verbal Comprehension	89	23	83–96
Perceptual Reasoning	92	30	85–100
Full-scale IQ	89	23	84–94

## Discussion and conclusions

There are only a few case reports about brain abscesses in EP infants [4–8]. To our knowledge, this is the first case report of an EP infant with a long- term follow-up. Brain abscesses were identified by MRI in this case and were treated by 7-week of systemic antibiotics and early surgical aspirations. With follow-up, MRI showed encephalomalacia in the frontal and parietal lobes of the right hemisphere and bilateral periventricular leukomalacia, but the girl had no obvious neurological deficits based on multiple aspects of examination.

With the development of neuroimaging, neurosurgery, and effective antibiotics, the mortality of brain abscesses has declined in children [2, 9–13]. However, rate of adverse neurological sequelae remains high [2, 9–12]. With increasing survival, more attentions are needed to improve the prognosis. Prognosis largely depends on early diagnosis and treatment [10]. Brain abscesses are found more frequently in cases of neonatal meningitis and septicemia [2], however, they can be diagnosed without meningitis and the positive rate of blood cultures was low [5, 14, 15]. This patient’s blood culture and cerebrospinal fluid examination were all negative. Furthermore, the lack of the evaluation of catheter-related bloodstream infection was our limitation in the case. Sundaram V. et al., proposed that serial cranial ultrasonograms may be required in septic neonates, to facilitate early identification of brain abscesses [14]. Repeated ultrasonograms performed at bedside should be routine procedure in septic neonates. In this case, brain abscesses were unexpected findings on MRI and serial brain ultrasound performed after the septic episode would have allowed an even earlier diagnosis. However, small abscesses and subdural collections may be missed by ultrasonography [16]. CT scan and MRI results are also important to make the diagnosis and to establish the treatment strategy.

This patient was treated with 7-week antibiotics and received surgical aspiration twice. The first aspiration was performed 9 days after the brain abscesses detected by MRI. Previous study has recommended an aggressive surgical approach of all abscesses larger than 2.5 cm in diameter, combined with 6–8 weeks of intravenous antibiotics [17]. Currently, the major surgical procedure used to treat them is drainage and aspiration, excision being of secondary importance [15]. Compared with aspiration, the scars of central nervous system and epilepsy are more common after excision [18]. Besides, early intervention of surgical procedure is essential to the good outcome [2, 10]. Delayed surgical drainage has high morbidity and mortality [10].

Previous studies showed that developing human brain possessed a superior capacity to reorganize after focal lesions [19–21]. Comparing to adult brain, reorganization in developing brain is often dramatically more effective and certain brain functions can primarily develop in atypical locations [21]. Guzzetta A. et al., showed that for the patients diagnosed with congenital hemiplegia, somato-sensory function was generally reorganized within the affected hemisphere [22]. In this case, MRI showed encephalomalacia in the frontal and parietal lobes of the right hemisphere and bilateral periventricular leukomalacia. Previous studies showed that if the abscess was in the parietal lobe, it led to hemiparesis and if the abscess was in the frontal lobe, it led to epilepsy [9, 23]. Nevertheless, the girl had no obvious neurological deficits. We presume the patient could have benefit from a reorganization of the neural network. But further methods are needed to study the reorganization in this patient.

In this case, we emphasize that using appropriate imaging techniques for early diagnosis of brain abscesses and that having appropriate therapies may improve the prognosis. Furthermore, it was a good case to demonstrate the possible neuroplasticity of brain in an EP infant.

## Data Availability

Not applicable.

## Abbreviations

EP: Extremely preterm
MRI: Magnetic resonance imaging
EEG: Electroencephalogram (EEG);
IQ: Intellectual quotient
NICU: Neonatal intensive care unit
N-CPAP: Nasal continuous positive airway pressure
PICC: Peripherally inserted central catheter
CT: Computed tomography
